# Complex signatures of selection for the melanogenic loci *TYR*, *TYRP1 *and *DCT *in humans

**DOI:** 10.1186/1471-2148-8-74

**Published:** 2008-02-29

**Authors:** Santos Alonso, Neskuts Izagirre, Isabel Smith-Zubiaga, Jesús Gardeazabal, José Luís Díaz-Ramón, José Luís Díaz-Pérez, Diana Zelenika, María Dolores Boyano, Nico Smit, Concepción de la Rúa

**Affiliations:** 1 Dept. Genetics, Physical Anthropology and Animal Physiology. University of the Basque Country. Barrio Sarriena s/n. 48940 Leioa, Bizkaia, Spain; 2 Dept. Zoology and Animal Cell Biology. University of the Basque Country. Barrio Sarriena s/n. 48940 Leioa, Bizkaia, Spain; 3 Dermatology Service. Cruces Hospital. Plaza de Cruces s/n, 48903, Cruces-Barakaldo, Bizkaia, Spain; 4 Centre National de Génotypage (CNG). 2 Rue Gaston Cremieux. CP 5721. 91057 Evry Cedex, France; 5 Dept. Cell Biology and Histology. University of the Basque Country. Barrio Sarriena s/n. 48940 Leioa, Bizkaia, Spain; 6 Department of Clinical Chemistry, L01-036 Leiden University Medical Center, Albinusdreef 2, 2333 AZ Leiden, The Netherlands

## Abstract

**Background:**

The observed correlation between ultraviolet light incidence and skin color, together with the geographical apportionment of skin reflectance among human populations, suggests an adaptive value for the pigmentation of the human skin. We have used Affymetrix U133a v2.0 gene expression microarrays to investigate the expression profiles of a total of 9 melanocyte cell lines (5 from lightly pigmented donors and 4 from darkly pigmented donors) plus their respective unirradiated controls. In order to reveal signatures of selection in loci with a bearing on skin pigmentation in humans, we have resequenced between 4 to 5 kb of the proximal regulatory regions of three of the most differently expressed genes, in the expectation that variation at regulatory regions might account for intraespecific morphological diversity, as suggested elsewhere.

**Results:**

Contrary to our expectations, expression profiles did not cluster the cells into unirradiated versus irradiated melanocytes, or into lightly pigmented versus darkly pigmented melanocytes. Instead, expression profiles correlated with the presence of Bovine Pituitary Extract (known to contain α-MSH) in the media. This allowed us to differentiate between melanocytes that are synthesizing melanin and those that are not. *TYR, TYRP1 *and *DCT *were among the five most differently expressed genes between these two groups. Population genetic analyses of sequence haplotypes of the proximal regulatory flanking-regions included Tajima's D, HEW and DHEW neutrality tests analysis. These were complemented with EHH tests (among others) in which the significance was obtained by a novel approach using extensive simulations under the coalescent model with recombination. We observe strong evidence for positive selection for *TYRP1 *alleles in Africans and for *DCT *and *TYRP1 *in Asians. However, the overall picture reflects a complex pattern of selection, which might include overdominance for *DCT *in Europeans.

**Conclusion:**

Diversity patterns clearly evidence adaptive selection in pigmentation genes in Africans and Asians. In Europeans, the evidence is more complex, and both directional and balancing selection may be involved in light skin. As a result, different non-African populations may have acquired light skin by alternative ways, and so light skin, and perhaps dark skin too, may be the result of convergent evolution.

## Background

The pigmentation of the human skin is the result of a complex process by which the pigmentary biopolymer, melanin, is produced and packed in the melanosome (a specialized organelle of melanocytes) and is distributed to the surrounding epidermal keratynocytes. Based mainly on the study of mouse mutants, a description has been made of over 100 loci that are involved in the pigmentary phenotype [1].

Although a lot of work has been done on the biochemistry and cytology of pigmentation, the evolutionary genetic history of this phenotypic trait has been less investigated. Initially, the observed correlation between ultraviolet radiation (UVR) and skin color [2], together with the geographical apportionment of skin reflectance among human populations in the major continental regions [3], suggest that the evolution of human skin pigmentation has been adaptive. Early evolutionary studies focused on *MC1R *diversity [4,5]. Although both these works observed a depletion of variability in Africans in comparison to Europeans and Asians, which was explained by purifying selection in Africans, the interpretation for the enhanced Euro-Asiatic diversity was different. Thus, while [4] claimed that *diversifying selection *has been responsible for the high diversity found in Eurasians, [5] invoked *functional relaxation*, and therefore neutral evolution, for this locus outside Africa. Phylogenetic comparison of *MC1R *primate sequences by [6] indicated that *MC1R *has been subject to purifying selection.

Other genes have been associated with the pigmentary phenotype, such as the *P *locus (OCA2) [7], *MATP *(alias *AIM1, SLC45A2*) [8] or *ASIP *[9,10]. More recently, it has been suggested that different genes may be responsible for the light pigmentary phenotypes observed in different non-African populations. Accordingly, a derived allele in a SNP within *SLC24A5 *correlates with lighter skin in Europeans, but not with light skin in East Asians [11]. Additional support for this point came from the work by [12], who used public SNP databases to single out *DCT *as a pigmentation gene candidate for recent positive selection in the Chinese only. In addition, [13] found that while *ASIP *and *OCA2 *might play a shared role in shaping light and dark pigmentation across the globe, genes like *SLC24A5*, *MATP *and *TYR *may have had a predominant role in the evolution of light skin in Europeans *but not *in East Asians. According to the authors, this provides compelling evidence that light skin has evolved independently in European and East Asian populations. Other genome-wide scans for genes under recent positive selection from ascertained SNPs claimed several pigmentation genes (*OCA2*, *MYO5A*, *DTNBP1 *and *TYRP) *as candidates for adaptive selection in Europeans only [14], whereas [15] suggested that protection against the damaging effects of UVR might hold an adaptive value in Africans.

However, most of the genetics of pigmentation is based on the mouse as a model organism. In contrast to humans, melanocytes in adult murine skin are generally confined mainly to hair follicles (also to the external ear and tail) [16]. It is therefore likely that some of the pigmentation genetics learnt from mice may not be directly applicable to humans. In this context, we have used Affymetrix U133A 2.0 microarray analysis to investigate the gene expression profiles of different human melanocyte cell lines. This is expected to provide us with a list of loci involved in human skin pigmentation, which can be used as candidate loci for evolutionary inference. Taking these results into account, we have resequenced between 4 to 5 kb of the proximal regulatory regions of three genes of interest, namely, *TYR*, *TYRP1 *and *DCT*. These genes form a homogeneous unit, as they belong to the same family and coordinate the production of melanin from tyrosine. Thus, the rate-limiting enzyme in melanogenesis is tyrosinase (EC 1.14.18.1) (the product of *TYR*), which catalyses the conversion of tyrosine into dopaquinone [17]. Tyrosinase activity is required for the synthesis of two types of melanins, *pheomelanins *(red to yellow melanins) or the more photoprotective *eumelanins *(brown to black melanins). Pheomelanogenesis seems to be the default pathway in the absence of MC1R signaling, with low tyrosinase activity and a high concentration of cysteine. Instead, eumelanin synthesis requires α-MSH binding to MC1R [18,19], which transcriptionally activates tyrosinase and upregulates tyrosinase-related protein-1 (EC 1.14.18, the product of *TYRP1*) and DOPAchrome tautomerase (EC 5.3.2.3; the product of the *DCT *locus, formerly *TYRP2 *or *TRP-2*). Other genes like *ASP*, the α-MSH antagonist, slightly reduce tyrosinase activity and produce an almost complete loss of *TYRP1 *and *DCT *expression, thereby decreasing eumelanin synthesis [20].

We have focused on the proximal regulatory region, as it has been proposed that variation at regulatory regions may account for intraespecific morphological diversity [21-25]. We therefore resequenced 116 human chromosomes from diverse geographical origin, including Africans, Europeans (plus European melanoma patients), Asians and Australian Aborigines, in search of diversity patterns that could help us reconstruct the evolutionary history of human skin pigmentation.

## Results and discussion

### Microarray experiments

Affymetrix U133a v2.0 microarrays were used to analyze five melanocyte cell lines from lightly pigmented donors and four from darkly pigmented donors, plus their respective unirradiated controls. Two additional cell lines from darkly pigmented donors did not yield RNA of sufficient quality for reverse transcription.

The first eigenvector for the SAM Pattern Discovery (unsupervised) analysis identified 5,404 probes as differentially expressed at an FDR of 4.65% (see Additional file 1). Unexpectedly, expression values did not cluster the cells into unirradiated versus irradiated melanocytes, or into lightly pigmented versus darkly pigmented melanocytes. Instead, we observed a differentiation between the M1 and M4 cells on the one hand (M1: lightly pigmented donor, irradiated and unirradiated; M4: darkly pigmented donor, irradiated and unirradiated), and the rest of the cell lines on the other. One possible explanation points to the different growth media used. However, a precise comparison of the media is difficult because providers do not always disclose the detailed formulation of the commercial media. With the information available, we can speculate on the absence of melanocyte mitogens BPE and/or PMA in the M1 and M4 cell cultures as a possible explanation. Both PMA and BPE could be boosting proliferation and melanogenesis in the "rest of the cells" group. In fact, both M1 and M4 proved to be very slow growing. In this regard, BPE usually contains α-MSH, which is a strong stimulator of melanogenesis. It has been shown that the induced release of α-MSH by UV light-irradiated keratinocytes (and to a much lesser extent, by irradiated melanocytes also) stimulates melanogenesis [26]. Along these lines, [27] reported also that human melanocytes cultured in media containing BPE do not respond to increasing concentrations of α-MSH, whereas the removal of BPE resulted in a significant reduction in melanocyte proliferation and melanogenesis, but restored responsiveness to melanotropins. However, for microarray experiments, the difficulty in growing melanocyte cultures without BPE may hamper the obtaining of the high cell numbers required. To confirm the effect of the presence/absence of BPE on the expression profiles of *TYR*, *TYRP1 *and *DCT*, we quantified by qPCR (see Material and Methods, Quantitative PCR section) the expression levels of these genes in one cell line that was grown in (a) Cascade growth medium 254 supplemented with 1% HGMS (includes BPE) vs. the same cell line grown in (b) Cascade medium plus PromoCell Supplement (no BPE). In this case all three genes suffered a reduction in their expression levels when grown under the second conditions (normalized ratios (conditions b)/(conditions a) *TYR*: 0.44 ± 0.09, *TYRP1*: 0.27 ± 0.05 and *DCT*: 0.05 ± 0.01) (3 replicates). We also investigated the effect of adding a known amount of BPE to the medium and thus we compared the expression levels of these when cell were grown in (c) PromoCell growth medium plus PromoCell Supplement vs. (d) PromoCell growth medium plus PromoCell Supplement and 0.2% (v/v) of a 13 mg/ml solution of BPE. Addition of BPE led to an increase of the expression level of all three genes (normalized ratios (conditions d)/(conditions c) *TYR*: 1.5 ± 0.22, *TYRP1*: 1.96 ± 0.26 and *DCT*: 1.93 ± 0.74) (2 replicates). These results indicate that in melanocytes, BPE has a biological effect on the expression of, at least, *TYR*, *TYRP1 *and *DCT*.

In any case, the homogeneity found in the gene expression profiles of human melanocytes grown in BSE-containing media suggests that melanocytes from both light and dark pigmentation donors may have the same genetic ability to produce melanin if subject to the same level of external (paracrine) signaling molecules, at least above a certain concentration.

Interestingly enough, however, Gene Ontology analysis of differentially expressed genes between these two groups indicates that the term "melanin biosynthesis from tyrosine" is a significant, non-redundant, "biological process" term (FDR p-value 0.0197). Genes belonging to this biological term are overexpressed in the "rest of the cells" group. Among the top over-expressed probes we can find those corresponding to *MLAN*-*A*, *TYR*, *TYRP1*, *DCT, GPR143, S100B, OCA2, SILV or SOX10*, and further down the list, *MITF, PAX3, KIT or SLC45A2 (MATP)*, genes known for their involvement in melanocyte function and development. *MC1R *is further down the list, but still shows an average log_2 _difference in gene expression of over 3 (overexpressed in the "rest of the cells" group). Rather unexpectedly, *SLC24A5*, a gene recently claimed as a major pigmentation gene in humans [11], does not appear in this list. Nevertheless, this list provides a good collection of candidate loci for further diversity analysis in human populations from which to obtain evolutionary inferences. Non-redundant GO terms (Biological processes) over-presented in the M1 and M4 cells group include: cell-adhesion (FDR p-value 1.3e-6), cell-cell signaling (FDR p-value 2.05e-5), inflammatory response (FDR p-value 2.05e-5), skeletal development (FDR p-value 2.55e-4), chemotaxis (FDR p-value 6.7e-4), morphogenesis (FDR p-value 1.6e-3), sensory perception (FDR p-value 3.9e-3), organelle organization and biogenesis (FDR p-value 6.5e-3), RNA processing (FDR p-value 1.4e-2), phosphate transport (FDR p-value 1.4e-2), cell surface receptor linked signal transduction (FDR p-value 1.9e-2), humoral defense mechanism (sensu Vertebrata) (FDR p-value 2.06e-2), reproductive organismal physiological process (FDR p-value 3.1e-2), DNA repair (FDR p-value 3.1e-2), cellular macromolecule metabolism (FDR p-value 3.2e-2), metal ion transport (FDR p-value 3.3e-2) and protein metabolism (FDR p-value 3.8e-2).

Based on this information, we decided to resequence the proximal regulatory regions of the melanogenic loci *TYR*, *TYRP1 *and *DCT *in search of evidence for selection on these genes. It is expected that resequencing, rather than typing previously ascertained SNPs, will provide unbiased information that will help us to more faithfully reconstruct the evolutionary history of these pigmentation loci.

### Population diversity and neutrality tests from sequence data

**Figure 1:**
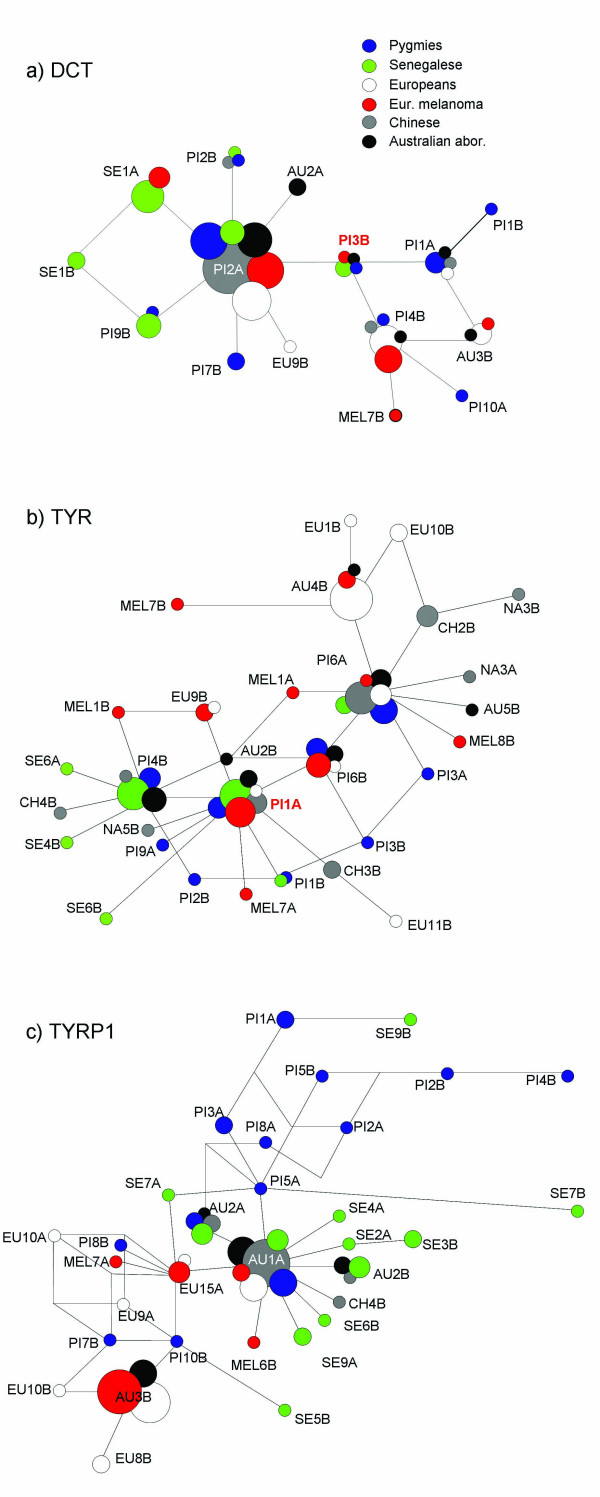
**Median-Joining network describing the genealogical relationships among the haplotypes found in the resequenced 5'-flanking regions of *TYR*, *TYRP1 *and *DCT***. Areas are proportional to absolute frequency. Blue circles: Pygmies; white circles: Europeans; red circles: European melanoma patients; gray circles: Chinese; green circles: Senegalese; and black circles: Australian Aborigines. The ancestral allele for *DCT *and *TYR *is indicated in red. The ancestral allele for *TYRP1 *could not be unequivocally identified, but most likely falls two mutational steps away from AU1A.

In the approximately 4 kb of the *TYR *upstream genomic region resequenced we have detected 20 SNPs in the global sample of 116 chromosomes. We have also detected two polymorphisms derived from runs of Ts at chr11:88548969 and chr11:88550172, one GA microsatellite between chr11:88549822.88550079, one polymorphic *Alu *insertion at chr11:88548585, which seems to belong to the Ya5 family, and three polymorphic indels, one of them involving 4 bp and two involving 2 bp. None of these non-SNP polymorphisms was considered for the diversity analysis, partly because of their different mutational nature to SNPs and partly because of the intrinsic difficulty in sequencing through some of these regions. Out of the above 20 SNPs, one falls within a putative CAAT box located about 200 bp upstream the translation initiation codon. None of them falls within other known *cis*-regulatory regions [23]. These polymorphisms assemble into 29 different haplotypes, whose genealogical relationships are shown in Figure 1.

In the approximately 5 kb of the *TYRP1 *upstream genomic region resequenced we have detected 29 SNPs. Polymorphisms derived from a run of Ts at chr9:12679614 and from a run of As at chr9:12680664 as well as a polymorphic rearrangement involving a 18 bp insertion found at chr9:12683765, were not considered in the analysis for the same reasons as above. None of the 29 SNPs fell within known *cis*-regulatory regions [23]. These polymorphisms assemble into 32 different haplotypes, whose genealogical relationships are shown in Figure 1. Haplotype frequencies and genotypes are available as supplementary material (see Additional file 2)

For the 4.1 kb region upstream from *DCT*, we have detected a total of 9 SNPs plus one indel (involving 1 bp), which was not considered for the sequence analysis for the same reasons as above. None of the SNPs falls within the known *cis*-regulatory regions located in the approximately 500 bp upstream of the transcription initiation point [23,28]. These polymorphisms assemble into 15 different haplotypes, whose genealogical relationships are shown in Figure 1.

Detected SNPs and indels have been submitted to dbSNP and will be publicly available in Build 130 (see Additional file 3).

After removing polymorphic positions, we estimated the divergence between a chimp sequence and the human sequence. The estimated divergence for *DCT *was 0.01 (95% C.I. 0.0074–0.0136); 0.0104 (95% C.I. 0.0075–0.0134) for *TYRP1 *and 0.0152 (95% C.I. 0.0114–0.0198) for *TYR*. These values fall within normal average neutral values [29] and suggest that no substantial evolutionary acceleration has occurred in these genomic regions since the human-chimp split.

Diversity measures for all samples are summarized in Table 1. Overall, both *DCT *and *TYRP1 *show higher haplotype diversity in the African populations analyzed, whereas *TYR *shows similar levels of haplotype diversity across populations.

**Table 1:** Diversity parameters and neutrality test for the resequenced regions of TYR, TYRP1 and DCT

	Pygmies	Senegal	Australian Ab.	Chinese	Europeans	Melanoma
**Number of segregating sites, S:**						
*DCT*	6	4	4	4	4	4
*TYR*	5	8	5	7	6	7
*TYRP1*	11	21	5	3	6	4
**Number of Haplotypes, h:**						
*DCT*	9	6	4	4	5	6
*TYR*	9	7	7	7	8	10
*TYRP1*	13	12	4	4	7	5
**Haplotype diversity, Hd (SD):**						
*DCT*	0.79 (0.09)	0.82(0.05)	0.66(0.12)	0.28 (0.13)	0.70 (0.07)	0.74 (0.07)
*TYR*	0.89 (0.04)	0.77 (0.06)	0.88 (0.06)	0.85 (0.06)	0.70 (0.1)	0.88 (0.05)
*TYRP1*	0.93 (0.04)	0.94 (0.03)	0.71 (0.08)	0.36 (0.13)	0.71 (0.08)	0.57 (0.12)
Nucleotide diversity, Pi (SD) (*10^-4^)						
*DCT*	3.8 (0.7)	3.1 (0.3)	3 (0.7)	1.2 (0.6)	3.5 (0.4)	3.5 (0.5)
*TYR*	5 (5)	3.8 (0.8)	4.9 (0.6)	5.5 (0.7)	3.8 (0.1)	5.4 (0.8)
*TYRP1*	7 (1)	6 (1)	4 (1)	1 (0)	4 (1)	2 (1)
Theta (per site) from S, Theta-W (SD^a^) (*10^-4^)						
*DCT*	4.1 (1.7)	2.7 (1.4)	3.1 (1.5)	2.8 (1.4)	2.7 (1.3)	2.7 (1.4)
*TYR*	3.8 (1.7)	6.1 (2.2)	4.3 (1.9)	5.3 (2)	4.5 (1.8)	5.3 (2)
*TYRP1*	6 (2)	12 (3)	3 (1)	2 (1)	3 (1)	2 (1)
**Average number of nucleotide differences, k:**						
*DCT*	1.574	1.253	1.231	0.489	1.446	1.421
*TYR*	1.832	1.4	1.802	2.032	1.403	1.989
*TYRP1*	3.268	2.963	1.890	0.389	1.827	1.289
**Tajima's D value**						
*DCT*	-0.221	0.321	-0.071	-1.638	0.892	0.754
*TYR*	0.917	-1.278	0.506	0.098	-0.458	0.027
*TYRP1*	0.192	-1.906	0.700	-1.447	0.341	0.416
**Tajima's D, p (D <= D obs)**						
*DCT*	0.39^b^(0.37)^c^	0.67^b^(0.59)^c^	0.52^b^(-)^c^	0.04^b^(0.03)^c^	0.84^b^(0.65)^c^	0.8^b^(0.62)^c^
*TYR*	0.86^b^(0.79)^c^	0.04^b^(0.02)^c^	0.73^b^(-)^c^	0.57^b^(0.39)^c^	0.33^b^(0.21)^c^	0.54^b^(0.38)^c^
*TYRP1*	0.64^b^(0.49)^c^	1e-3^b,d^(1e-3)^c,d^	0.8^b^(-)^c^	0.09^b^(0.04)^c^	0.67^b^(0.44)^c^	0.69^b^(0.5)^c^
**DHEW (HEW) p-values**						
*DCT*	0.19 (0.26)	0.68 (0.73)	0.20 (0.22)	4e-3^e ^(5e-3^f^)	0.63 (0.32)	0.57 (0.20)
*TYR*	0.75 (0.81)	0.33 (0.43)	0.62 (0.69)	0.27 (0.12)	0.1 (3e-3^f^)	0.52 (0.63)
*TYRP1*	0.87 (0.91)	0.61 (0.72)	0.76 (0.82)	0.28 (0.33)	0.4 (0.03)	0.44 (0.03)

^a ^assuming free recombination.

^b ^using the standard neutral model in DnaSP.

^c ^using a demography-corrected neutral model.

^d ^significant after multiple test correction, at an FDR of 5%.

^e ^significant after multiple test correction, at an FDR of 8%.

^f ^significant after multiple test correction, at an FDR of 6%.

Among the non-African populations, the Chinese sample shows a substantial drop in diversity for both *DCT *and *TYRP1*. This drop in diversity is accompanied by significantly negative Tajima's D values for both *DCT *and *TYRP1*, although these values become non-significant after multiple-test correction (data not shown). DHEW and HEW tests are also significant for *DCT *in the Chinese (even after multiple-test correction, at an FDR of 8% and 6%, respectively) (Table 1). These results suggest possible positive selection acting on alleles of these loci in the Chinese, in particular for *DCT*.

The European samples, however, show positive (although non-significant) Tajima's D values for *DCT *and *TYRP1*. Excluding population structure, which is unlikely to be substantial in our European sample, positive Tajima's D values can be obtained when some kind of diversity-increasing selection is operating [30]. Analysis of the HapMap data confirms the existence of SNPs (like rs9301959, rs7990565 or rs4773797) with higher than expected heterozygosity (p < 0.01) in the extended *DCT *region in Caucasians. This observation is compatible with selection by overdominance. Europeans and European melanoma patients show some evidence for selection at *TYRP1 *(HEW test, see Table 1), but this is not significant after multiple-test correction. Caucasians share their major *TYRP1 *haplotypes, AU1A and AU3B, with Australian Aborigines. The AU1A allele seems to pre-date the out-of-Africa expansion, since all African and non-African populations analyzed also share this haplotype. This suggests that this haplotype has little to do with pigmentation differences across populations. As regards AU3B, the recent claim that aboriginal Australians descend from the same African emigrant group as all other Eurasians [31] suggests an age for this shared allele of at least 50,000–70,000 years. Thus, despite the relatively high frequency of AU3B in Europeans, given the pigmentary differences between Australian Aborigines and Europeans and the lack of strong evidence for selection in them, it seems unlikely that this haplotype is associated with a light skin phenotype, at least in a simple way. In Europeans, only *TYR *shows some evidence for selection but only for the HEW test (significant after multiple-test correction at an FDR of 6%). This is most likely due to the fact that HEW is more powerful than DHEW in detecting ongoing or recently completed selective sweeps [32]. The contrasting patterns between Tajima's D and HEW in *TYR *might indicate a partial sweep at this locus in the European (healthy) sample. This signature is not observed however in the sample of European melanoma patients. Interestingly, European melanoma patients show a frequency for AU4B that is markedly lower than that for healthy Europeans (Fischer's exact test p-value: 0.0025). However, this observation should be considered only as exploratory, as it is not our purpose in this work to conduct a formal test of association to melanoma.

For the African populations analyzed (Pygmies and Senegalese, both dark-skinned populations), the networks observed in Figure 1 suggest that the main factor responsible for the lack of strong negative Tajima's D values in the Pygmy sample is population structure. In fact, our Pygmy sample is composed of both Biaka and Mbuti Pygmies, who have been described as "substantially different genetically" [33]. This observation can explain the positive Tajima's D values found for the Pygmy sample [34].

In contrast, the Senegalese record a highly significant negative Tajima's D value for *TYRP1 *(significant even after multiple-test correction, at an FDR value of 5%), which is suggestive of selection. The observation that neither DHEW nor HEW are significant for the African populations (Table 1) is however consistent, as DHEW and HEW are expected to have much less power than Tajima's D to detect selection after fixation of the advantageous allele when ρ is no greater than θ [32], which is the case for the Senegalese *TYRP1 *diversity. It may be argued that the simulation parameters inferred from the Yoruba are not applicable to other African populations. In an attempt to offset the effects of a possible population expansion on Tajima's D p-value observed for the Senegalese, and in the absence of a precise demographic model for this population, we have run further simulations assuming for the Senegalese the demographic model used previously for the Europeans. This is expected to provide a reasonably conservative correction. Under this demographic scenario, the p-value for Tajima's D in the Senegalese is approximately 0.027. Thus, we are inclined to believe that the negative Tajima's D in the case of the Senegalese *TYRP1 *diversity reflects the signature of an old selective process.

Lastly, the lack of signal in Australian Aborigines can be explained by the effect of drift: if the population that left Africa and colonized Australia was relatively small and relatively constant, whereby the effect of drift overcame the effect of selective pressure, then this signal of selection may have been lost.

### Other Selection tests based on the HapMap project data

In order to detect selection in more recent evolutionary time windows [35], we have used the SNP frequency information available in the HapMap dataset to perform, firstly, a scan for SNPs with significant pairwise F_ST _values across these loci, and secondly, a search for longer than expected haplotypes within populations by means of the EHH test [36] (see also Material and Methods).

F_ST _comparison is useful for detecting selection in a time window of less than 50,000 to 75,000 years [35]. After multiple-test correction, several SNPs were found to be positive across *DCT *and *TYRP1 *(including the flanking regions) for the HapMap Chinese+Japanese vs. HapMap Caucasians pairwise comparison (Table 2). Overall, our results agree with those by [12], although in our case, multiple-test correction makes non-significant additional pairwise comparisons that [12] found significant for their uncorrected F_ST _tests.

**Table 2:** HapMap genotyped SNPs showing significant F_ST_^a^

Locus	SNP ID	**Position**^**b**^	Gene region	**F**_**ST **_**CEU vs ASI**^**c**^
*TYRP1*	rs13293905	Chr9:12675943	5' flanking region	0.679
	rs10756393	Chr9:12682252	5' flanking region	0.558
	rs2762462	Chr9:12689776	intron	0.638
	rs2762463	Chr9:12691897	intron	0.671
	rs2733832	Chr9:12694725	intron	0.581
	rs2733833	Chr9:12695095	intron	0.661
	rs2075509	Chr9:12695219	intron	0.553
	rs2209277	Chr9:12696236	intron	0.671
	rs683	Chr9:12699305	3' UTR	0.644
	rs2762464	Chr9:12699586	3' UTR	0.652
	rs910	Chr9:12700035	3' UTR	0.671
	rs1063380	Chr9:12700090	3' UTR	0.671
	rs2733835	Chr9:12702157	3' flanking region	0.581
	rs12379024	Chr9:12707405	3' flanking region	0.599
	rs10491744	Chr9:12710106	3' flanking region	0.599
*DCT*	rs1325611	Chr13:93892386	intron	0.607
	rs1407995	Chr13:93894014	intron	0.594
	rs2031526	Chr13:93898842	intron	0.607
	rs3782972	Chr13:93901047	intron	0.641
	rs6492706	Chr13:93937385	5' flanking region	0.468

^a ^Multiple-test corrected significant F_ST _values at an FDR of 5% (see Material and Methods). A total of 97 SNPs were analyzed.

^b ^Coordinates from HapMap Data Rel. 21a/PhaseII, Jan07 on NCBI B35 assembly, dbSNP b125.

^c ^only CEU vs. ASI show multiple-test corrected significant F_ST _values. CEU: HapMap North European Caucasians; ASI: HapMap Chinese plus Japanese.

**Figure 2:**
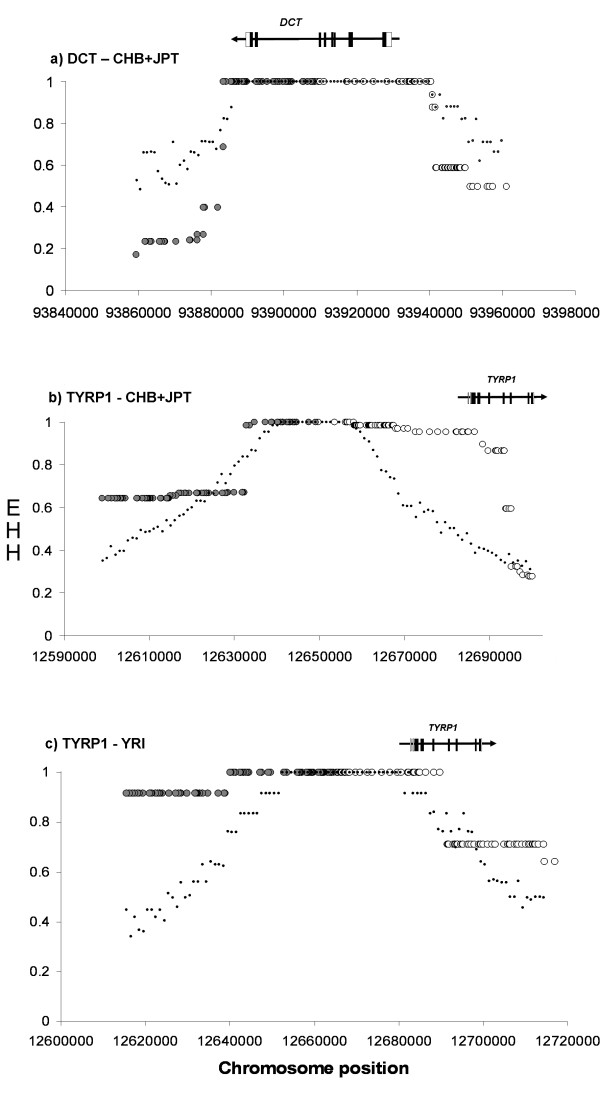
**Extended Haplotype Homozygosity (EHH) tests**. After scanning the HapMap data with *Sweep *(see Methods) candidate core haplotypes were tested for the significance of their extended haplotype homozygosity (EHH). Circles represent the observed EHH values extending about 50 kb from the core haplotype in both directions, white for 3' and gray for 5'. Dots represent the corresponding 95% upper percentiles of the simulated EHH distributions. The approximate location of the lociwith respect to the core haplotypes is indicated on top: coding exons in black, UTRs in white, and the intervening lines represent the introns. The arrows indicate the direction of transcription. a) EHH for *DCT *in the HapMap Chinese+Japanese population; b) EHH for *TYRP1 *in the HapMap Chinese+Japanese population and c) EHH for *TYRP1 *in the HapMap Yoruba population.

EHH tests can be useful to detect signatures of partial selective sweeps in a time window of less than 30,000 years [35]. We observed haplotypes with longer than expected EHH in the HapMap Chinese+Japanese sample for *DCT *and *TYRP1*, and in the HapMap Yoruba for *TYRP1 *(Table 3, Figure 2).

**Table 3:** Haplotypes with longer than expected homozygosity (EHH test) for *DCT *and *TYRP1*.

Locus	Core SNPs IDs	**Chr position**^a^	Core haplotype	**frequency**^c^			**pop**^d^
				CEU	ASI	YRI	
*DCT*	rs9516418	Chr13:93909510	TCA	5%	18%	0%	ASI
	rs9584234	Chr13:93909660	(101)^b^				
	rs12877248	Chr13:9390983					
							
*TYRP1*	rs10809814	Chr9:12649098	AA	5%	71%	19%	ASI
	rs4741242	Chr9:12649691	(10)^b^				
							
*TYRP1*	rs10960751	Chr9:12665264	TGA	0%	0%	20%	YRI
	rs10960752	Chr9:12665284	(001)^b^				
	rs932761	Chr9:12665380					

^a ^Coordinates from HapMap Data Rel. 21a/PhaseII, Jan07 on NCBI B35 assembly, dbSNP b125.

^b ^Between brackets, ancestral/derived coding of haplotypes; 0: ancestral, 1: derived

^c ^CEU: HapMap North European Caucasians; ASI: HapMap Chinese+Japanese; YRI: HapMap Yorubas.

^d ^Population in which the core haplotype shows longer than expected EHH at a significance value of 0.05 (see Methods).

Using a different long-range haplotype statistic for *DCT*, [12] suggested the existence of a core SNP at rs2031526 whose bidirectional breakdown of haplotype homozygosity of the derived allele "A" evidenced selection. Our EHH approach (see Material and Methods), however, did not identify this SNP as a strong candidate core, perhaps because our procedure to declare significance (see Material and Method) is more stringent than that used by [12]. Instead, our *DCT *core haplotype "TCA", which is located around 11 kb upstream from rs2031526, overlapping an intronic conserved region in mammals, actually identifies a subset of the rs2031526 "A" haplogroup.

The core SNPs for *TYRP1 *HapMap Yoruba fall within a conserved region (77% identity with the mouse orthologous sequence) that contains at least one potential transcription factor binding site for SOX10 (a known melanogenesis enhancer) and is placed less than 5 kb upstream of a putative *TYRP1 *enhancer sequence [37].

The relatively low frequencies of *DCT *core haplotype "TCA" in the Chinese+Japanese and of *TYRP1 *core haplotype "TGA" in the Yoruba (Table 3) suggest that we are detecting the signature of selection in young haplogroups only. EHH is expected to fail to detect the signature of selection in older haplotypes because of the decay of LD with time. However, at least in the case of *TYRP1*, the fact that both Tajima's D and EHH detect selection may indicate that the environmental pressure was probably old, and either continued to act for some time after the origin of this "TGA" allele and then ceased or is still ongoing.

Finally, the core SNPs for *TYRP1 *in the HapMap Chinese+Japanese fall astride a LTR (MER52A, family ERV1) and a LINE (L1MA2) element, around 15 kb upstream from the aforementioned core SNPs for *TYRP1 *in the HapMap Yoruba. Complete homozygosity for a core haplogroup "AA" extends well into the 5'-flanking region of the gene, and seems to constitute a major subset of allele AU1A. Its high frequency (see table 3) and long EHH correlates with the (weak) signal of selection detected by Tajima's D in the Chinese *TYRP1 *sequence diversity.

The proximity of the two core haplotypes for *TYRP1 *observed in two different populations, in combination with the long EHH that extends into the flanking region of the locus, and the evidence for selection on these two populations from the Tajima's D test, point to a region distally upstream of *TYRP1 *as the target region for selection on *TYRP1 *diversity.

## Conclusion

It is well-established that dark skin is photoprotective (see [38] and references therein), but whether this photoprotective role represents an evolutionary advantage is less clear. Alternative mechanisms to explain a possible adaptive advantage of dark pigmentation involve *concealment *[39] or the protection of *folate *from UVR-induced photolysis [40]. However, in the light of the apparent vagaries that occur in the pigmentation of mammalian skin, including primates, others [41] suggest that dark skin may have outlived its usefulness. Selective mechanisms for light skin involve the facilitation of vitamin D3 synthesis [40].

The ability to detect selection from diversity patterns depends on many aspects, such as the demography of the population under study, the mutation and recombination rates of the locus, the dominance and selection coefficients of the advantageous allele, whether the advantageous allele has reached fixation (and how long ago) or not, and whether the favorable allele arose from a single mutation at the time of the selective pressure or was favored from standing variation [42]. Thus, we have used a battery of different tests to enhance the ability to detect selection, and we have been able to show the signature of directional selection particularly on *TYRP1 *in Africans, and on *DCT *and *TYRP1 *in Asians. We should bear in mind that the constitutive pigmentation of the skin is not *exclusively *dependent on melanin production. Other factors, such as the distribution of melanins within melanosomes, the melanosome secretion rate by melanocytes, the rate of melanosome ingestion by keratynocytes and the distribution of melanosomes within keratynocytes, are also major determinants of skin pigmentation ([43] and references therein). The question of how these mechanisms can be functionally linked to the observed diversity patterns in the melanogenic loci studiedcannot be answered with the present data. However, the observed patterns of diversity do seem to support the idea that selection has had a role in the evolutionary history of melanogenesis.

As regards the nature of selection, the resequencing analysis did not detect any polymorphism in the known proximal regulatory-elements that could be associated with pigmentary differences. However, judging by the localization of the core SNPs identified by the EHH tests it seems likely that, at least in the case of *TYRP1*, distal regulatory regions (see [37]) are the targets of selection. Selection at the level of gene expression for *TYRP1 *would be compatible with the observation that the most darkly pigmented individuals seem to have more TYRP1 protein per melanocyte, which has been claimed to be at least partly responsible for the greater tyrosinase activity and melanogenesis reported for dark skin [44]. In addition, it seems likely that non-directional selection is acting too. For instance, overdominance seems compatible with the diversity patterns observed for *DCT *in Europeans. Similarly, the high frequency of the ancestral *TYR *haplotype within the Senegalese suggests that purifying selection at this locus may have been of some importance within Africa, although the large number of *TYR *mutations associated with oculocutaneous albinism 1 (OCA1) (OMIM#230100) suggest that *TYR *may be a gene under purifying selection in all populations. Interestingly, Stokowski et al. [45], by means of a multistage genomewide association study, have recently found that polymorphisms in *TYR *(along with *SLC24A5 *and *SLC45A2*) show significant associations with skin-reflectance measurements in a South Asian population. Whether this is a particular characteristic of this population remains to be explored, but altogether, this suggests that a number of different selective regimes may have interacted in a complex way in the evolution of melanogenesis. As a result, the lighter skin pigmentation phenotype in Europeans and East Asians may have been acquired by alternative mechanisms and thus, as previously suggested [11-13,15], light skin would be the result of convergent evolution. Similarly, the diversity profile of Australian Aborigines suggests that this may also be the case for dark skin.

## Methods

### Cells and cell cultures

All melanocytes used were obtained from human skin. Melanocyte line M0202 was obtained from a lightly pigmented donor by one of us (NS) in the lab. Melanocytes M1 ("Caucasian", 1.5 years old, male donor) and M4 ("Negroid", 4 years old, male donor) were purchased from Promocell (Heidelberg, Germany). The 19-HEM cell line (lightly pigmented) was purchased from Gentaur (Belgium), and NHEM cells (two lightly pigmented and three darkly pigmented) were purchased from Cascade Biologics (Nottinghamshire, UK).

Cell cultures were maintained in an incubator at 37°C and 5% CO_2_. Melanocyte culture conditions were as indicated by the suppliers. Briefly: a) M0202 cells were initially grown in FETI medium: H-10, 2% FCS, 1% Ultroser G (BioSepra, Ciphergen, France), 4 ng/ml bFGF, 2 ng/ml endothelin-1, 5.3 nm TPA, 0.05 mM IBMX, but were later grown in H-10 supplemented with HEPES 6 mM, 5% FBS and MelanoMax (Gentaur), which presumably contains TPA, CT and contains BPE at 40 μg/ml (C. Stefanidis, Gentaur; personal communication); b) M1 and M4 melanocytes were cultured in melanocyte growth medium M2 (Promocell). M2 is a serum-free medium, without PMA (TPA) or other tumor promoting or toxic agents, consisting of a basal medium plus a Supplement Mix. The detailed formulation has not been disclosed by the supplier, but it seems that no BPE is present in this growth medium (Ute Liegibel, PromoCell, personal communication). c) 19-HEM melanocytes were grown in H-10 supplemented with HEPES 6 mM, 5% FBS and MelanoMax (Gentaur); d) NHEM melanocytes culture medium was Cascade Biologics Medium 254 supplemented with 1% HMGS (containing BPE, FBS, bovine insuline, bovine transferrin, bFGF, hydrocortisone, heparin and PMA) (Cascade Biologics).

The culture medium was changed every two days until the culture was approximately 80–90% confluent, and everyday thereafter.

M0202, HEM and NHEM melanocytes were passaged (split 1:3) routinely every 10 or 11 days, or harvested when they had reached confluency. The growth rate for M1 and M4 melanocytes was slow, and passaging (1:2) or harvesting at confluency was performed routinely after 2 to 3 weeks. All melanocytes used were from passage 5 to 15, and all were from normal non-transformed primary cell culture isolates.

### Irradiation

Subconfluence cultures were irradiated once a day for 5 consecutive days with UVA+B (50 mJ/cm^2^:25 mJ/cm^2^) light in an ICH2 photoreactor (LuzChem, Canada) at 37°C. These doses assumed an absorbance of the plastic flask of approximately 5% for UVA and 11% for UVB (flasks were opaque to UVC), estimated from spectrophotometer absorbance readings at 255 nm, 305 nm and 360 nm of cuvette-size splinters of the flasks. By trials with murine melanoma cells (B16F10), this dose was shown to have no effect on cell viability. In order to prevent the generation of toxic metabolites, the culture medium was replaced by PBS with magnesium and calcium immediately before irradiation. After irradiation, PBS was replaced again by the culture medium. Irradiation control cultures were subject to the same procedure, except that they were covered by aluminum foil during irradiation. Cultures were harvested 24 h after the last irradiation dose.

### Microarray gene expression

Immediately after harvesting, cells were resuspended in lysis buffer. Total RNA was obtained following the supplier's protocol (Ambion's total RNA extraction kit), including DNAse treatment. cDNA was synthesized from 2 μgr of total RNA using the Affymetrix One-Cycle cDNA Synthesis Kit and following the Affymetrix Expression Analysis Technical Manual. From this cDNA, cRNA was synthesized using the Affymetrix IVT Labeling Kit, which was then purified with the Affymetrix GeneChip Sample Cleanup Module. The purified cRNA (15 μgr) was fragmented and hybridized to Affymetrix U133A 2.0 arrays using standard Affymetrix protocols. A total of 18 microarrays from 9 irradiated cell lines (5 from lightly pigmented donors and 4 from darkly pigmented donors) plus their corresponding unirradiated control cultures were analyzed.

### Microarray Data Analysis

Raw data were log_2 _transformed and quantile normalized using DNAMR v1.1 [46] for R (2.4.1). The Pattern Discovery option of SAM software v3.0 [47] was used to analyze the normalized data. Gene Ontology analysis was done using FatiGO [48].

### Quantitative PCR

To demonstrate that the media composition, in particular the presence/absence of BPE, affects the expression levels of *TYR*, *TYRP1 *and *DCT *we purchased a new melanocyte cell line from Cascade Biologics (lightly pigmented) and grew this cell line in Cascade growth medium 254 supplemented with 1% HGMS (Cascade + for short). We sub-cultured and propagated the cells for approximately 3 weeks (three passages). Three days after the third passage we generated 4 subcultures of the same cell line. These 4 subcultures were from now on grown in 6 different media: Medium 1: Cascade +; Medium 2: Cascade medium, PromoCell Supplement (no BPE); Medium 3: PromoCell growth medium plus PromoCell Supplement. Medium 4: PromoCell growth medium plus PromoCell Supplement, plus 0.2% (v/v) of a 13 mg/ml solution of BPE. Cells were grown in these media for four days to allow some adaptation to the new media. Media were refreshed every two days.

After this time, we extracted total RNA from each subculture (Ambion). cDNA was synthesized using the Invitrogen SuperScript First-Strand synthesis system for RT-PCR kit, and then, we quantified the expression of *TYR*, *TYRP1 *and *DCT *for each of these subcultures using the BIO-RAD iQ SYBR green Supermix system in combination with a BIO-RAD iCycler machine. For mRNA quantification the following primers were used: *TYR*: 5'-AGAATGCTCTGGCTGTTTTG-3' and 5'-TCCATCAGGTTCTTAGAGGAGACAC-3'. For *TYRP1*: 5'-CATGCAGGAAATGTTGCAAGAG-3' and 5'-AGTTTGGGCTTATTAGAGTGGAATC-3'; For *DCT*: 5'-TATTAGGACCAGGACGCCCC-3' and 5'-TGGTACCGGTGCCAGGTAAC-3'. For normalization *GADPH *was used; primer: 5'-CCTGTTCGACAGTCAGCC-3' and 5'-CGACCAAATCCGTTGACTCC-3'. In all cases, annealing temperatures were fixed at 56°C. For *DCT*, Mg^2+ ^concentration was increased in 1 mM above the standard reaction conditions.

### DNA samples and Resequencing

We have resequenced the following in 116 human chromosomes: a) 4.1 kb of 5' *DCT*, including 187 bp of the first intron, the first CDS plus the 5'-UTR and 3,204 bp of the upstream flanking sequence; b) approximately 4 kb of 5' *TYR*, including 17 bp of the first intron, the first CDS plus the 5'-UTR and 2,773 bp of the upstream flanking sequence; and c) approximately 5 kb of 5' *TYRP1*, including 433 bp of the first intron, 47 bp of the second intron, the first CDS plus the 5'-UTR and 4,200 bp of the upstream flanking sequence. Sample individuals come from diverse geographical origins and include: 20 chromosomes from Biaka and Mbuti Pygmies (DNA purchased from the European Collection of Cell Cultures, ECACC), 20 chromosomes from Senegalese individuals resident in Spain, 42 European (N. Spain) chromosomes (including 20 chromosomes from melanoma patients), 20 Asian chromosomes including Chinese samples from Coriell Cell Repositories and Chinese residents in Spain, and 14 chromosomes from Australian Aborigines (DNA purchased from the ECACC).

DNA was PCR amplified in overlapping ~1 kb long segments and these were resequenced using BigDye 3.1 chemistry and ABI PRISM 3730 and ABI 310 DNA analyzers. Sequences were edited with Genalys v3.3.45a (M. Takahashi). Primers and PCR conditions are available on request. Sequences have been deposited in GenBank, accession numbers DQ821585-DQ821701 for *DCT*, EF675246-EF675361 for *TYR *and EF675362-EF675477 for *TYRP1*.

### Estimation of haplotypes

To solve the haplotypes phase, we first run PHASE [49]. For those pairs of SNPs that did not reach a PHASE probability greater than 0.95, we solved their phase experimentally by ARMS-PCR and/or cloning (using the TOPO-TA kit from Invitrogene) plus resequencing.

### Population parameters and neutrality tests

After removing polymorphic positions in humans, divergence (K) between a chimp sequence and a human sequence was estimated using K-estimator 6.0 [50]. Initially, population diversity parameters and neutrality statistics like Tajima's D [29] were obtained by means of DnaSP 4.1 [51]. These tests were corrected for demography as specified below. HEW and DHEW tests [32] were carried out using software kindly provided by Kai Zeng.

### Optimization of demographic parameters

To correct for demography in the coalescent neutral simulations of the neutrality tests (excluding HEW and DHEW), we optimized the fit between pairwise F_ST _distributions obtained from real genomic data from the three major geographical human groups in the HapMap project, and those F_ST _distributions from coalescent simulations obtained varying the demographic parameters. The optimization criterion was the p-value of the Kolmogorov-Smirnov D statistic between the real and simulated F_ST _distributions. For the real neutral distribution of the F_ST _statistic [52], we used that obtained previously in [15] in which we selected 43 regions distributed across the autosomal genome that belong to broader regions of low gene density, and which are at least 150 kb away from the closest exon. Each of these regions spanned an average of 1.96 Mb, and in total they account for 84.3 Mb. For each region we downloaded the SNP frequency information available from the HapMap browser (data Rel #20/phase II on NCBI B35 assembly, dbSNP b125) for the 3 major populations (Caucasians: 153,339 SNPS, Yorubas: 123,798 SNPs and Chinese: 33,190 SNPs). We further filtered the number of SNPs to include only those SNPs that: a) were at least 100 kb away from each other, b) have been genotyped in all three populations and c) at least one of the three major populations had a minor allele frequency (MAF) higher than 0.1. A final list of 546 SNPs satisfied these criteria. We used the *ms *program [53] for the simulations with 3 populations, with sample sizes equal to those in the HapMap population (120 chromosomes for the African population, 120 for Caucasians and 90 for Asians). As starting points in our simulations to optimize demographic parameters, we used those values described in [54]. Simulations were fixed on one segregating site. These SNPs were matched for a MAF of 0.1 in at least one population.

The mean and 95% upper limits (between brackets) of the observed F_ST _distributions were [15]: Caucasians-Chinese: 0.08 (0.33); Caucasians-Yorubas: 0.14 (0.47) and Chinese-Yorubas: 0.16 (0.45). Final optimized values were obtained in the simulations under the following conditions: we used an ancestral population size of 24,000 for the African population (population 1) and 7,700 for both Asians and Caucasians (populations 2 and 3, respectively), with a migration rate matrix M_ij _= {0, 0.05, 0.4, 0.1, 0 3, 0.8, 2.5, 0} for *i *and *j *values from 1 to 3. Looking back in time, we assumed two bottlenecks with instant population reduction, each followed by a population fusion: one approximately 40,000 years ago, in which the Chinese population reduced its size to approximately one sixth. About 2,000 years after this episode, the Chinese population fuses with the European population. Assuming a generation time of 20 years, this represents an F value of 0.04 for this bottleneck. A second population bottleneck takes place about 90,000 years ago. On this occasion, the Eurasian population suffers a reduction in size to one sixth of its previous size. About 10,000 years after this bottleneck (F = 0.21), the Eurasian population fuses with the African population. The mean and 95% upper limits (between brackets) of the simulated F_ST _distributions were: Caucasians-Chinese: 0.08 (0.31); Caucasians-Yorubas: 0.15 (0.44) and Chinese-Yorubas: 0.15 (0.45). The optimized, simulated F_ST _distribution and the real distribution recorded Kolmogorov-Smirnov D values of 0.0356 for Caucasians vs. Asians (*p*-value 0.891), 0.0748 for Caucasians vs. Africans (*p*-value 0.104), and 0.0441 for Asians vs. Africans (*p*-value 0.683). As both simulated and real data are not statistically different, we used those demographical parameters used in the simulations for the subsequent neutrality tests.

### Correction for demography in the neutrality tests

These demographic and genetic parameters were subsequently used in further simulations for estimating the critical points in Tajima's D. These simulations also allowed us to obtain the distribution of Tajima's D under the genetic and demographic parameters described above.

### Extended Haplotype Homozygosity (EHH) test

For the EHH test, we initially used Sweep 1.0 [55] to scan and select the core haplotypes. We initially focused on those haplotypes that showed both substantial frequency and high EHH values from the core SNPs, in both 5' and 3' directions. By means of a Perl script, we then calculated the EHH values as in [26] for a region extending about 50 kb from the closer core SNP. To test the significance of the test, we ran coalescent simulations using the population and demographical parameters described above for the F_ST _distributions, but in this case several aspects are different from our previous approach [15]. First, in this case we included the variable recombination rate information obtained from the HapMap webpage for the particular region tested. In each case, we obtained a discrete number of recombination rate classes using the following approach: for each region we obtained the average and standard deviation (sd) of the distribution of recombination rates obtained from the HapMap link. All recombination values greater than the average plus 2 sd were considered outliers. If these outlier regions were consecutive, a local average was calculated; otherwise, a single outlier value was assigned for that region flanked between the previous and the next recombination values. We then excluded these outliers and repeated the process. In this second round, recombination rates that were higher than the new global average plus 1 sd were considered again as a class each, except if they were consecutive, in which case a local average was estimated. The average of the rest of recombination rates was considered as the background recombination rate, which was used as a reference to estimate the relative intensity of recombination for all other recombination classes.

For the coalescent simulations with heterogeneous recombination rates, we used *msHOT *[56], a modification of [53] coalescent-based program (*ms*) for simulating genetic variation data for a sample of chromosomes from a population. Second, in order to obtain a null distribution that reflects a neutral scenario for the chosen core haplotypes, we proceeded as follows: a) in the simulations, the "core" was the set of the first *n *SNPs, where *n *is the number of SNPs in the original core for each case. We then discarded those simulations that did not result in a number of distinct haplotypes (as defined from the simulated *core *set of SNPs) identical to that observed in the HapMap data using the corresponding observed *core *SNPs; b) one of the simulated haplotypes had to match in frequency (allowing for a 2% difference) our observed core haplotype being tested, and in addition, it had to show the same ancestral/derived states for the SNPs composing the core. For the latter, ancestrality was obtained by comparing the corresponding orthologous regions from the chimpanzee genome sequence and the *Macaca mulatta *genome sequence obtained from the UCSC genome browser [57] or the Ensembl genome browser [58]; c) for each set of simulations that fulfilled these conditions (we typically ran the program until we obtained about 500 simulations satisfying all the conditions), we obtained the 95% upper percentile of the expected EHH distribution for each of a series of consecutive 1 kb-long windows spanning the region. We finally declared a core haplotype as under selection if the distance for which the EHH values were equal to 1 for the SNPs in the HapMap data was longer than the distance observed from the 95% upper percentile distribution of the EHH values obtained in the simulations.

This approach offers several advantages over other methods currently being used to detect selection. For instance, it allows for a direct comparison with a "neutral" null distribution. In contrast, null distributions obtained from genomic regions are not representative of a homogeneous neutral scenario, but rather the result of heterogeneous evolutionary processes. In addition, it allows statistical inference even when no alternative haplotypes for the same core SNPs are available in order to obtain relative EHH values, as done in other approaches. Finally, ancestral-derived state information and information on the core haplotypes frequency can be incorporated, which helps fine-tune the nature of the elements being compared (observed and simulated data).

### Multiple testing corrections

To control for multiple testing in the F_ST _tests, we used the approach by [59], which sets the false discovery rate (FDR) at a level α by ranking the initial *p *values in ascending order *P*_(1) _≤ *P*_(2) _≤ ... ≤ *P*_(m)_, with *m *being the number of tests, and then by specifying *P*_(i) _≤ αi/*m *as the point below which there is no rejection at an FDR of α.

### Genealogical relationships among haplotypes

Graphical representations of the genealogical relationships among haplotypes were estimated by the Median-Joining (MJ) algorithm implemented in Network 4.1.1.2 [60]. When feasible, the ancestral haplotype was inferred using parsimony by comparison to the chimp and rhesus macaque sequences.

### Test for overdominance

To evaluate the possibility of overdominance (heterozygote advantage), we scored the ratio of "observed heterozygosity" to "expected heterozygosity" for single SNPs. Observed heterozygosity was estimated by counting heterozygote individuals in the HapMap data set for each SNP in question. The expected heterozygosity was estimated by calculating gene diversity from allele frequencies. Significance for ratio values greater than 1 was obtained by simulation using *ms *[53].

## Authors' contributions

SA and CR conceived and designed the study. SA analyzed the microarray data, devised the Perl scripts, and conducted most of the population genetic analyses. SA and CR wrote the first draft of the manuscript.

NI did the bulk of the resequencing work and haplotype inference.

DZ contributed significantly to the resequencing work.

ISZ, MDB and NS took responsibility for the cell cultures

JLDP, JLDR and JG participated in the design of the study, obtained the DNA from the melanoma patients and devised the irradiation experiments.

All authors reviewed the manuscript. All authors read and approved the final draft.

## Supplementary Material

Additional file 1 containing the analyzed Affymetrix U133A v2 gene expression normalized values for the 9 melanocyte cell lines (both control and irradiated with UV Light). Click here for file

Additional file 2 containing allele frequencies and genotypes. Click here for file

Additional file 3 containing the SNPs and indels submitted to dbSNP. Click here for file
